# Measuring Production Efficiency and Structural Factors in Sheep Farming: Empirical Evidence from North-Eastern Anatolia, Türkiye

**DOI:** 10.3390/ani16193131

**Published:** 2026-10-06

**Authors:** Ayşe Karadağ Gürsoy, Bahri Karlı

**Affiliations:** 1 Department of Agricultural Economics, Faculty of Agriculture, Iğdır University, Iğdır 76000, Türkiye; 2 Department of Agricultural Economics, Faculty of Agriculture, Isparta University of Applied Sciences, Isparta 32000, Türkiye; bahri.karli@isparta.edu.tr

**Keywords:** sheep, technical efficiency, stochastic frontier, data envelopment analysis, factor analysis, Türkiye

## Abstract

This study evaluates the technical efficiency and structural factors influencing sheep farming enterprises operating in the TRA2 sub-region (Ardahan, Kars, Ağrı, and Iğdır provinces) of North-Eastern Anatolia, Türkiye. Primary data were collected from 138 sheep farms selected through a stratified random sampling method. The technical efficiency of the enterprises was evaluated using a dual methodological framework combining Data Envelopment Analysis (DEA) and Stochastic Frontier Analysis (SFA). Input-output variables integrated into the efficiency frontier model included the gross production value of sheep farming as the output, alongside roughage expenditure, concentrate feed expenditure, family labor, and veterinary/medicine costs as input variables. The empirical findings yielded a mean technical efficiency score of 0.657, supported by a Likelihood Ratio (LR) test statistic of 20.569 (*p* < 0.01), confirming significant technical inefficiency across the sampled farms. To identify the socio-economic determinants of efficiency, a Tobit regression analysis was performed; while operator age exhibited a positive relationship, sheep farming experience, household size, and formal education duration showed negative associations with efficiency scores, though none reached statistical significance. Additionally, Factor Analysis was employed to uncover the underlying structural multidimensional factors shaping sheep production dynamics in the region. The findings highlight key managerial and operational constraints, providing actionable insights for policymakers to promote sustainable smallholder livestock development in mountainous agro-ecological zones.

## Simple Summary

The purpose of this study was to determine the efficiency of sheep breeding enterprises in the TRA2 sub-region. The TRA2 region was selected as the study area due to its geographical location and characteristics, which make it an important region for sheep farming in Türkiye. The primary data of the study comprised information obtained through face-to-face questionnaires from 138 sheep breeding enterprises in the Ardahan, Kars, Ağrı, and Iğdır provinces within the TRA2 region. To measure the efficiency levels of the enterprises, five variables were included in the research: sheep breeding gross production value (USD/LU), forage amount (kg/LU), concentrate feed amount (kg/LU), family labour (hours/LU), and veterinary medicine expenses (USD/LU). It was determined that a decrease in the frequency of feeding increased the efficiency of the enterprises.

## 1. Introduction

Small ruminants constitute a vital component of global livestock production, particularly in developing countries and marginal production systems. Beyond their low initial costs and manageable size, they are uniquely suited for smallholders and efficiently convert forage under temperate, arid, or semi-tropical conditions [1]. Furthermore, their remarkable adaptability enables them to survive and produce in harsh environments, such as arid, high-altitude, or extremely cold regions. Small ruminants are better adapted to hot environments than large ruminants and have a superior ability to survive, produce, and reproduce under harsh climatic conditions [2]. This adaptive capacity allows them to mitigate the impacts of environmental variability while providing multipurpose value through meat, milk, and fibre, particularly serving as critical livelihood assets for women and youth in dryland communities [3]. A balanced and adequate diet for human health enhances the quality of life. The livestock sector is an indispensable industry for providing the essential nutrients required by the body. Furthermore, the livestock sector is important in terms of providing employment to many industries, such as the feed industry, meat and meat products industry, leather industry, and pharmaceutical industry. The small ruminant sector is of paramount importance as it meets the demand for animal products and simultaneously serves as a source of livelihood for small family enterprises [4,5,6]. A significant branch of small ruminant farming is sheep breeding. Sheep were among the first livestock animals to be domesticated by humans. The point at which humans began spinning sheep’s wool into yarn and using it marks an important turning point in human development. In this respect, sheep farming can be considered the beginning of industry for humanity. Sheep breeding, which is thought to be one of the earliest livelihoods of humans, has largely maintained its place in the agricultural economy from past to present and has become a major source of production and employment worldwide. Sheep are found in tropical countries and the poles, in hot climates and cold, and in desert and humid regions. There are more than 800 sheep breeds of various sizes, shapes, types, and colours across the globe. There are very few countries where sheep breeding is not practised. The reason for the high prevalence of sheep farming worldwide is that small ruminants easily adapt to harsh environmental conditions and the feeding regimes of their respective regions [7,8]. The versatile benefits of sheep farming constitute its most significant advantage. In sheep farming, meat and wool yield different streams of income due to their distinct characteristics in terms of production and utilisation. Since wool is a storable product, it can be kept for as long as desired and supplied to the market when needed. Moreover, lambs obtained from sheep become marketable from approximately the fifth month onwards, providing a rapid generation of income [7]. According to data from 2024, there are approximately 1.36 billion head of sheep worldwide. China holds the highest share of the global sheep population at 13.43%. China is followed by India in second place with a share of 8.41%, and Australia in third place with a share of 5.82%. Türkiye ranks seventh in the world in terms of the number of sheep, accounting for a 3.24% share [9]. Sheep breeding has been practised in Türkiye for many years. It is a particularly important activity for rural areas. Although sheep breeding in Türkiye is an economic activity, it is also a part of cultural life. As is the case globally, sheep breeding is carried out in almost every region of Türkiye [10,11]. Considering its geographical structure and vast pastures, Türkiye possesses an appropriate environment and potential for sheep farming. Sheep breeding stands out for its ability to adapt to due to seasonally insufficient pastures and unfavourable climatic conditions. In Türkiye the most important way to utilise areas where crop production cannot be conducted is their enabling the efficient utilization of low quality pastures and crop residues by small ruminants and the production of various animal products. The vast majority of pastures in Türkiye have low productivity and are more suitable for small ruminant farming [12,13]. Compared to most other agricultural production alternatives, sheep breeding does not require high specialisation, and a low amount of capital is sufficient [14]. The TRA2 Region, located in the Eastern Anatolia Region, is one of the 26 sub-development regions in Türkiye and consists of four provinces. The TRA2 Region, comprising the Ardahan, Kars, Ağrı, and Iğdır provinces, is an important region where sheep farming is conducted in Türkiye due to its geographical location and characteristics. Considering the climatic and ecological conditions of the TRA2 Region, including its high altitude, extensive meadow-pasture lands and plant diversity, pastoral livestock farming remains the predominant production system. Consequently, sheep farming plays a pivotal socio-economic role within the region’s agricultural structure. Sheep breeding occupies an important place in the Turkish economy in terms of meat, milk, wool, and leather production. Sheep farming in Türkiye is an important source of livelihood, particularly in the Eastern Anatolia and Southeastern Anatolia regions [6]. While the TRA2 Region, which is the research area, held 8.68% of Türkiye’s sheep population in 2006, it possesses 7.58% of Türkiye’s sheep population as of 2026. The share of the TRA2 region within Türkiye has gradually decreased over the 20-year period since 2006. This study was conducted to identify the determinants underlying technical inefficiency in order to assess the extent to which sheep farming enterprises in the TRA2 region utilize their existing resources effectively. In this regard, the research contributes to the existing literature in two fundamental ways. Methodologically, the study integrates non-parametric (Data Envelopment Analysis) and parametric (Stochastic Frontier Analysis) efficiency frontiers with multivariate Factor Analysis and Tobit regression, thereby enabling a comprehensive assessment of both technical efficiency and farm-specific latent structural factors. From an empirical perspective, the study presents the first multi-stage efficiency and structural benchmarking framework for sheep farming in the TRA2 sub-region of Northeast Anatolia, offering unique insights and policy recommendations for the pasture-based livestock economy in developing regions.

## 2. Materials and Methods

### 2.1. Study Area

The primary data of the study comprised first-hand information obtained through face-to-face questionnaires from enterprises engaged in sheep breeding activities in the Ardahan, Kars, Ağrı, and Iğdır provinces located in the TRA2 region. The questionnaire surveys were conducted in 2024, and data regarding the 2020 production period were obtained. In addition, the secondary data of the research were sourced from statistical records and publications of the Turkish Statistical Institute, the Food and Agriculture Organisation (FAO), the Ministry of Agriculture and Forestry, and the Provincial Directorates of Agriculture and Forestry in the TRA2 region. Furthermore, various national and international studies and reports related to the subject were utilised.

### 2.2. Data Collection

In order to better represent the population, interpret the obtained findings more accurately, and establish more balanced farm-size groups, the Neyman method, a stratified sampling technique, was used to determine the number of sample enterprises to be surveyed [15,16]. Accordingly, the number of samples to represent the population was calculated using Equation (1).

(1) $$n=\frac{{\left(\sum \mathrm{Nh}\times \mathrm{Sh}\right)}^{2}}{N^{2}\times D^{2}+\sum \mathrm{Nh}\times Sh^{2}}$$

n: sample size,

N: total number of units in the population (population size),

Nh: number of units in the hth stratum,

Sh: standard deviation of the hth stratum,

Sh^2^: variance of the hth stratum,

D^2^: d^2^/z^2^,

d^2^: permissible margin of error from the population mean (5%),

z^2^: value from the distribution table for the permissible confidence limit (95%).

According to the determined sample size, the enterprises were randomly selected from the population. The enterprises engaged in sheep breeding were divided into four strata based on the frequency distribution, taking into account the number of sheep. Equation (2) was used to distribute the enterprises into the strata [15,17].

(2) $$n_{h}=\frac{N_{h}\times S_{h}}{\sum N_{h}\times S_{h}}\times n$$

Nh: number of samples selected for each stratum (sample size per stratum),

n: total sample size.

The number of samples representing the population was calculated as 138 at a 95% confidence interval and a 10% margin of error. The enterprises engaged in sheep breeding were divided into four strata according to their livestock holdings. Stratum I represents enterprises with 1–100 head of sheep, Stratum II represents 101–250 head, Stratum III represents 251–750 head, and Stratum IV represents enterprises with 751 or more head of sheep (Table 1).

In the distribution of the sample size across the provinces, the allocation was carried out by taking into account the share held by each province within the research region (Table 2).

The data from the research region were obtained through questionnaires via face-to-face interviews with the enterprises engaged in sheep breeding. Before data collection, ethical approval was obtained from the Scientific Research and Publication Ethics Committee of Isparta University of Applied Sciences (Decision No: 176/03). A pilot survey was first administered using the prepared questionnaire form. Based on the results of the pilot survey, the final version of the questionnaire form was completed, and subsequently, the field study was conducted.

### 2.3. Data Analysis

Efficiency allows for the determination of where and in what position enterprises stand within the current competitive environment of the sector in which they operate and demonstrates the extent to which enterprises can produce output with the inputs they possess [18]. Through the measurement of efficiency and productivity, the success levels of enterprises can be evaluated. As a pivotal metric in agricultural economics, efficiency measures a farm’s proficiency in converting a given input combination into maximum feasible output [19,20].

#### 2.3.1. Efficiency Analysis

While often perceived as synonymous, efficiency and productivity represent fundamentally different analytical dimensions and calculation metrics. The former provides an objective measurement of input-output transformation dynamics, while the latter reflects a qualitative assessment of goal achievement. Mathematically defined as the ratio of outputs generated per unit of input consumed, productivity operates in tandem with efficiency. Nevertheless, explicitly disentangling these two interconnected concepts remains a persistent ambiguity in empirical studies [21,22,23,24].

##### Efficiency Measurement Methods

Efficiency measurements assume that the production function of enterprises is completely efficient. Since the production function of enterprises can never be known in reality, Farrell [20] proposed estimating this function by using enterprise data comparatively. According to Farrell [20], the measurement of an enterprise’s efficiency under a given technology should be conducted for two purposes: technical efficiency (TE) and allocative efficiency (AE). Together, these two efficiencies yield total economic efficiency. Technical efficiency refers to the potential of a firm to achieve the maximum output with its existing input set. Allocative efficiency, on the other hand, indicates the success of a firm in using inputs in the most appropriate proportion with its own cost structure and production technology.

Technical efficiency is the production of the maximum possible output by utilising inputs in the most efficient manner, or the use of the minimum possible input to obtain the current output without compromising quality. The success of an enterprise in producing the maximum possible output by utilising its input combination in the most optimum way is defined as technical efficiency, whereas the success in utilising the input combination in the most appropriate proportion by considering input and output prices or costs is defined as allocative efficiency. Economic efficiency (EE) is obtained through the joint evaluation of these two efficiency types [18,25,26].

EE = TE × AE(3)

TE = EE/AE(4)

AE = EE/TE(5)

Efficiency measurement methods in the agricultural sector are divided into two groups: parametric and non-parametric methods, oriented towards inputs and outputs. Data Envelopment Analysis (DEA) from non-parametric methods and Stochastic Frontier Analysis (SFA) from parametric methods are the two most frequently used approaches in measuring efficiency [27,28].

- Data envelopment analysis

Data Envelopment Analysis (DEA) was initially developed by Charnes [29] in 1978 to measure the relative efficiency of economic decision-making units producing similar goods or services, and it is a linear programming-based method for production frontier estimation [30]. DEA stands as a widely adopted non-parametric approach specifically designed to evaluate production efficiency in complex settings featuring multiple inputs and outputs that cannot be aggregated into unified totals. The core principle of efficiency estimation in DEA relies on the quotient of weighted outputs relative to weighted inputs. This mathematical relationship yields a comprehensive efficiency index for multi-factor decision-making units, expressed through the following formulation [19,31,32,33].

(6) $$\mathrm{Efficiency}=\frac{\mathrm{Weighted}\;\mathrm{Sum}\;\mathrm{of}\;\mathrm{Outputs}}{\mathrm{Weighted}\;\mathrm{Sum}\;\mathrm{of}\;\mathrm{Inputs}}+\frac{U1Y1j+U2Y2j+\ldots +\mathrm{UkYkj}}{V1X1j+V2X2j+\ldots +\mathrm{VmXmj}}$$

Uk: weight of the kth output,

Ykj: amount of the kth output of the jth unit,

Vm: weight of the mth input,

Xmj: amount of the mth input of the jth unit.

- Stochastic frontier analysis

Stochastic Frontier Analysis posits that an enterprise operates under a maximum attainable output ceiling for any specified combination of inputs. This methodology relies on farm-level input-output observations to construct an empirical production frontier [31,34]. Among the parametric techniques for efficiency measurement, Stochastic Frontier Analysis is the most frequently used method. SFA is also known as the econometric approach in the literature. In this approach, a functional relationship is established between explanatory variables such as inputs, outputs, and environmental factors occurring during production, and variables such as cost, profit, and production.

Stochastic Frontier Analysis (SFA), on the other hand, is a method developed by Aigner et al. [35], Battese and Corra [36], and Meeusen and Van Den Broeck [37] that constructs an efficient production frontier using econometric methods. The stochastic frontier production function approach is based on the assumption that some units do not utilise their resources efficiently. These units operate below the production frontier defined by the best production technology. In this approach, a random error—namely, the error term—is included [38].

According to Aigner et al. [35], the stochastic frontier production function is defined as follows;

Yi = Xi,β + (Vi − Ui)  i= 1, 2, 3, ….,N(7)

Yi: production (output) of the ith decision-making unit,

β: vector of parameters to be estimated for the (Kx1) dimensional inputs,

Xi: (K + 1) dimensional row vector of inputs,

Vi: random errors, random factors beyond the control of the enterprise, and other omitted inputs,

Ui: non-negative random variable representing technical inefficiency.

The technical efficiency of the sheep enterprises was estimated using Stochastic Frontier Analysis via Equation (8).

(8) $$\mathrm{TEt}=\frac{\mathrm{Yt}}{\mathrm{Yt}*}$$

TEt: technical efficiency of the ith decision-making unit,

Yt: production amount per unit area of the ith decision-making unit,

Yt*: potential production amount of the i-th decision-making unit per unit area estimated from the production function.

In this study, the Stochastic Frontier Analysis (SFA) was specified using a Cobb–Douglas type production function to model the functional relationship between inputs and sheep production value.

#### 2.3.2. Tobit Regression and Inefficiency Factor

While the values of dependent and independent variables are known in linear regression models, in some cases, the values of these variables may be censored or truncated [39]. Contrary to these models, the Tobit model is utilised in cases where certain values of the dependent variable cannot be observed. In Tobit models, dependent variable values can be censored or truncated from above or below [40].

The standard equation of the Tobit regression model is given below [41].

(9) yi=yi*,if yi*>00,if yi*≤0yi*=xi′β+ei

xi: value of the independent variable,

yi: value of the bounded (censored) dependent variable,

β: coefficient to be estimated,

ei: error term.

#### 2.3.3. Factor Analysis

Factor analysis can be defined, in its most basic form, as the explanation of the variance in a certain number of dependent variables through a smaller number of independent variables or factors [42,43]. Although factor analysis is the name given to a class of multivariate statistical methods whose primary objective is data reduction and summarisation, it mostly analyses the relationships among a large number of variables and subsequently explains these variables through general, fundamental dimensions or factors. Factor analysis is a multivariate statistical analysis that presents data in a meaningful and summarised manner by considering the relationships between data points [44]. Furthermore, each factor addressed in this method can also be viewed as a dependent variable that is a function of the original observed values [45].

There are many forms of the general factor model, with common factor analysis and component factor analysis being the most frequently used. The choice of the factor model is determined based on the objective of the research. Although the mathematical model of factor analysis resembles a multiple regression equation, each variable considered is expressed as a linear combination of factors that cannot actually be observed [46].

Numerous different options were tested until the results of the factor analysis were obtained, and four factors consisting of 14 variables with a high communality element in sheep enterprises were achieved. In the factor analysis conducted using the SPSS 21.0 software, the widely used Principal Component Analysis (PCA) model and the Varimax extraction method were utilised.

In this research, the applicability of factor analysis was tested using the Kaiser–Meyer–Olkin (KMO) and Bartlett’s test of sphericity. Communality, which is one of the most crucial factors in determining variables, indicates the representativeness of the variables. The closer this value is to 1.00, the more sound the variable selection is considered to be [47,48].

All monetary variables used in this study were converted from Turkish Lira (TRY) to United States Dollars (USD) using the official average USD/TRY exchange rate for the period of February 2020–January 2021 published by the Central Bank of the Republic of Turkey (1 USD = 7.16 TRY) [49].

## 3. Results

The variables used in calculating the efficiency scores of the enterprises were determined by considering the inputs extensively used in the technical analysis of sheep production in the region, those expected to have the greatest impact on yield, and the inputs intensively utilised by the producers.

In the research, five variables were included in the efficiency model, with sheep gross production value (USD/LU) as the dependent variable, and the quantity of roughage (kg/LU), the quantity of concentrated feed (kg/LU), family labour (hours/LU), and medication costs (USD/LU) as the independent variables. In the examined farms, the gross production value per livestock unit, serving as the dependent variable, was determined to be an average of 1431.83 USD, with a minimum GPV of 140.20 USD and a maximum GPV of 4236.65 USD. Regarding the independent variables, the averages were determined as 1689.16 kg for roughage quantity, 574.18 kg for concentrate feed quantity, 168.80 h for family labor, and 33.10 USD for veterinary/medication costs (Table 3).

The mean technical efficiency scores of the enterprises were calculated as 0.573 (SFA) and 0.657 (DEA) (Table 4). Under both methods, the highest technical efficiency was observed in Strata I, while the lowest was recorded in Strata III. The overall mean allocation efficiency and economic efficiency scores were 0.351 and 0.228, respectively, showing a clear upward trend from Strata I to IV [50]. Overall, to achieve full economic efficiency, the enterprises need to reduce their input costs by an average of 77.20%.

Analysis of the efficiency score distributions for Stratum I enterprises reveals that for parametric technical efficiency (SFA), the majority of enterprises (45.45%) were concentrated in the 0.501–0.750 score range, with no fully efficient enterprises observed (Table 5). Under non-parametric technical efficiency (DEA), 31.82% of the enterprises achieved full efficiency (1.000). Regarding allocative efficiency, 95.45% of enterprises fell into the lowest score range (0.001–0.250), while for economic efficiency, all enterprises (100.00%) were located in this lowest bracket, with neither category producing fully efficient enterprises.

Examining the distribution of efficiency scores for Stratum II enterprises, technical efficiency under SFA concentrated mainly in the 0.501–0.750 range (48.00%), with no fully efficient enterprises observed (Table 6). Under DEA technical efficiency, 44.00% of enterprises fell within the 0.501–0.750 bracket, while 12.00% (3 enterprises) achieved full efficiency (1.000). For allocative and economic efficiencies, the vast majority of enterprises were clustered in the lowest score category (0.001–0.250), representing 76.00% and 84.00%, respectively, with no enterprises exceeding the 0.751 threshold or reaching full efficiency in these two dimensions.

Evaluation of the efficiency score distributions for Stratum III enterprises shows that technical efficiency under SFA was concentrated mainly in the 0.251–0.500 (38.30%) and 0.501–0.750 (34.04%) ranges, with no fully efficient enterprises observed (Table 7). Under DEA technical efficiency, 40.43% of enterprises fell into the 0.251–0.500 bracket, while 17.02% (8 enterprises) achieved full efficiency (1.000). For allocative efficiency, enterprises displayed a more balanced dispersion across the middle ranges (0.251–0.750, accounting for 61.70%), whereas economic efficiency remained predominantly clustered (61.70%) in the lowest range (0.001–0.250). Neither allocative nor economic efficiency categories produced fully efficient enterprises.

Evaluation of the efficiency score distributions for Stratum IV enterprises (N = 44) shows that under parametric technical efficiency (SFA), the highest proportion of enterprises (40.91%) achieved high efficiency scores within the 0.751–0.999 bracket, though none reached full efficiency (Table 8). Under non-parametric technical efficiency (DEA), 22.73% of enterprises (10 enterprises) achieved full efficiency (1.000). For allocative efficiency, enterprises were primarily concentrated in the 0.501–0.750 (40.91%) and 0.251–0.500 (34.09%) ranges, whereas economic efficiency was mostly clustered in the 0.251–0.500 range (56.82%). No enterprises exceeded the 0.751 threshold or achieved full efficiency in the economic efficiency dimension.

Technical efficiency scores were specified as the dependent variable in the Tobit regression and inefficiency models, while producer age, sheep farming experience, education duration, household size, and daily feeding frequency were incorporated as independent variables (Table 9). Descriptive statistics indicate that the producers had a mean age of 51.20 ± 11.34 years (range 27–77), sheep farming experience of 17.44 ± 8.40 years (range: 1–50), and education level of 5.04 ± 1.96 years (range 1–11). The average household size was 6.67 ± 1.85 persons (range 2–9), and the mean daily feeding frequency was 2.62 ± 0.53 times (range 2–5).

The relationship between socio-economic variables and technical efficiency was determined using the Tobit regression analysis. The number of feedings in the enterprise was estimated to have a statistically significant effect at the 1% level. It can be stated that a decrease in the number of feedings would increase the technical efficiency score. In the Tobit regression model, although farmer age had a positive coefficient, and sheep farming experience, household size, and education duration had negative coefficients, no statistically significant relationship was determined between these demographic/socioeconomic variables and efficiency scores (*p*>0.05) (Table 10).

Whether the Ordinary Least Squares (OLS) or Maximum Likelihood (ML) estimation method should be applied in the Stochastic Frontier Analysis (SFA) was evaluated using the generalized one-sided likelihood ratio (LR) test [51].

LR = −2{In[LR(H_0_)] − In[LR(H_1_)]} (10)

where ln[*L*(*H*_0_)] represents the log-likelihood value of the OLS model (null hypothesis) and ln[*L*(*H*_1_)] represents that of the ML model (alternative hypothesis). The calculated LR statistic of 20.659 confirmed the superiority of the ML formulation over the OLS estimation, justifying the choice of the ML method for the SFA.

The inefficiency effects model examined the impact of socio-economic variables—specifically producer age, sheep farming experience, education duration, household size, and daily feeding frequency—on enterprise efficiency (Table 11). Among these, feeding frequency was the only variable displaying a statistically significant relationship, exerting a negative effect on technical efficiency (positive on inefficiency) at the 10% level. Conversely, while experience and age showed negative trends and household size and education showed positive trends with technical inefficiency, none reached statistical significance. Finally, the estimated gamma (*γ*) parameter of 0.949 (*p* < 0.01) demonstrates that 94.9% of the total variability in enterprise output was attributable to technical inefficiency rather than random shocks.

In classical economic studies, it is assumed that all producers operate efficiently. However, this assumption is not realistic. For this reason, the coefficients obtained from the inefficiency model were utilised in the analysis of the production function [52]. In the present study, the gross value of production (GVP) of sheep farming was the dependent variable, while roughage, concentrate feed, family labour, veterinary medicine cost, and the number of sheep constituted the independent variables. The veterinary medicine cost variable, which represents one of the major expenses in sheep farming, and the number of sheep were found to have a negative effect on the sheep GVP, and this relationship was statistically significant at the 10% level (Table 12).

The model calculated as a result of the Cobb–Douglas analysis is structured as follow.

$$Y=6.731X_{1}^{0.047}X_{2}^{0.025}X_{3}^{0.195}X_{4}^{0.136}X_{5}^{0.280}$$

The factors influencing sheep farming in the enterprises surveyed within the TRA2 region were determined via factor analysis, and several indicators regarding the variables associated with the calculated factors are presented (Table 13).

In this study, the applicability of factor analysis was determined using the Kaiser-Meyer-Olkin (KMO) measure of sampling adequacy and Bartlett’s test of sphericity. The KMO value was calculated as 0.759 (75.9%). Since this value is greater than 0.500, the dataset was determined to be suitable for factor analysis. Furthermore, Bartlett’s test was statistically significant (*p* less than 0.01). Accordingly, because correlation exists among the variables, the dataset is appropriate for factor analysis (Table 14).

**Figure 1:**
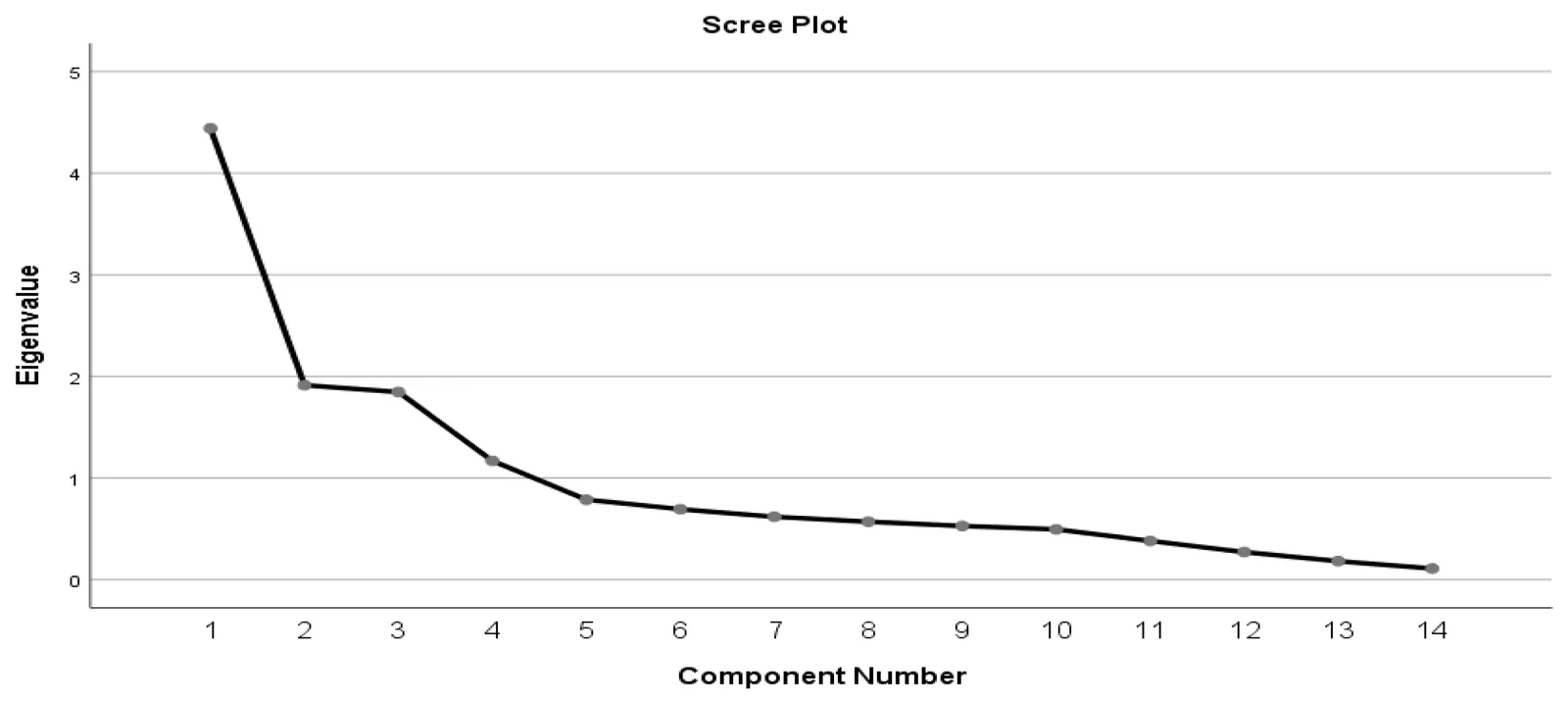
Scree plot of the factor analysis.

To determine the number of factors, a scree plot was utilized. In the scree plot, the *X*-axis represents the factor numbers, while the *Y*-axis represents the eigenvalues. The plot illustrates the four factors with eigenvalues greater than 1.00, which are estimated to influence the sustainability of sheep farming (Figure 1).

According to the statistical results of the initial solution of the factor analysis, the statistics regarding the variance explanation rates of the four factors with eigenvalues greater than 1.00 were examined. The four identified factors explain 66.92% of the total variance. The first factor accounts for 31.72% of the total variance, followed by the second factor at 13.67%, the third factor at 13.19%, and the fourth factor at 8.35% (Table 15).

It is observed that these variables, which are expected to be above 0.50 in the factor analysis, range between 0.518 and 0.801. In this respect, the common variance shared by the variables with the other variables is sufficient (Table 16).

The variables in the analysis were determined using the varimax rotation technique. The factor loadings, which represent the correlation coefficients between the variables used in the analysis and their associated factors, are presented in this matrix. In the rotated factor matrix, factor loadings are expected to be 0.30 or higher, while coefficients of 0.50 and above are classified as highly satisfactory [44,53,54]. Accordingly, based on the results of the factor analysis applied to determine the factors affecting sheep farming in the TRA2 region, the values range between 0.352 and 0.886 (Table 17).

According to the results of the varimax rotation method, Factor 1 explains 31.72% of the total variance, followed by Factor 2 (13.67%), Factor 3 (13.19%), and Factor 4 (8.35%).

## 4. Discussion

### 4.1. Data Envelopment Analysis Results

The technical efficiency (TE) score of the sheep farming enterprises in the research area was found to be 0.657. It was determined that these enterprises operate below the efficiency frontier. According to the findings obtained, among the studies conducted in Türkiye, Yılmaz [16] determined the average technical efficiency of enterprises in the Western Mediterranean region to be 0.77. It was stated that the higher rate of inefficiency found in the present study compared to the aforementioned study stems from regional and structural differences.

When the international literature is examined, a study conducted on sheep farming enterprises in Cyprus by Sokratous et al. [55] found the average technical efficiency to be 0.844. In a study conducted in Greece, this value was determined as 0.76 [56]. Another study in Spain reported that although the average technical efficiency was 66%, 74% of the surveyed enterprises operated at medium and low technical efficiency rates [57]. Considering these studies, the efficiency results indicate that there is a higher potential in terms of technology adoption and herd management.

### 4.2. Tobit Regression Analysis Results

According to the results of the study, it was determined that the frequency of feeding has a statistically significant effect at the 1% level. This negative relationship indicates that increasing the daily feeding frequency reduces technical efficiency, likely due to additional labor demands and potential feed wastage in stall-feeding practices. A similar inverse relationship between intensive feeding practices and technical efficiency in small ruminant systems has been documented in previous empirical studies [58].

A positive relationship was identified between farmer age and efficiency; however, this relationship was not statistically significant. When similar studies in the national literature are examined, Gül et al. [59] determined a positive relationship between education level and efficiency, but they did not find this to be statistically significant. In contrast, Dalgıç [60] found that efficiency increased as the education level reached 8 years and above, and this effect was statistically significant. Yılmaz [16] found a statistically significant and positive relationship between the feeding system, supplementary feeding status, and inefficiency.

Although sheep farming experience, household size, and farmer education level had negative directions, no statistically significant relationship was found between these variables and efficiency scores. Accordingly, there was no statistically significant relationship between the efficiency of sheep farming in the enterprises and sheep farming experience, household size, farmer age, and farmer education level. When international literature is reviewed, a study indicated a negative relationship between education level and efficiency [61].

### 4.3. Stochastic Frontier Analysis Results

As a result of the generalized likelihood ratio (LR) test conducted to determine the estimation method of the frontier model, the LR statistic was calculated as 20.569. When the coefficients of the production function are examined, the coefficients for the variables of medicine input (0.136) and the number of sheep (0.280) were found to be positive and statistically significant at the 10% level. According to the Cobb–Douglas production function, this indicates that a 10% increase in the number of sheep in the enterprise would lead to a 2.8% increase in production. When the technical inefficiency factors constituting the second part of the model are analyzed, the coefficient for the daily frequency of feeding variable was found to be 0.740 and statistically significant at the 10% level. In the Battese and Coelli [62] model, a positive coefficient represents an inefficiency-increasing (efficiency-decreasing) effect. Accordingly, as the daily frequency of feeding increases in the enterprises, technical inefficiency increases—in other words, technical efficiency decreases. This situation may stem from the fact that irrational, frequent feeding habits fail to optimize labor and feed costs. According to the model, it was determined that 94.9% of the variance is caused by technical inefficiency.

Külekçi and Bayram [63] reported in their study that a 10% increase in inputs would result in a less than 10% increase in live weight gain. In a study conducted in the United Arab Emirates, Fathelrahman et al. [64] stated that 56% of the variance in sheep farming was due to technical inefficiency.

## 5. Conclusions

This study evaluated the technical and economic efficiency of sheep farming enterprises in the TRA2 region of Türkiye, identifying key factors influencing productivity and enterprise performance. The empirical findings reveal that sheep breeding enterprises in the region operate with substantial technical inefficiencies (mean TE of 0.657 under DEA and 0.573 under SFA), with economic efficiency lagging even further behind technical efficiency. While non-parametric estimates demonstrate that full efficiency is attainable—particularly in larger enterprise strata—parametric models highlight a widespread systemic inefficiency across all farm sizes. A critical insight from both the Tobit regression and SFA inefficiency models is the significant negative impact of feeding frequency on technical inefficiency, indicating that excessive or unstandardized feeding routines increase labor and input costs without yielding proportional output gains. Furthermore, the functional analysis underscores that major expenditure items, such as veterinary medicine costs and larger flock sizes, negatively affect Gross Production Value (GPV), reflecting mismanaged input allocation and overstocking pressures. The factor analysis further corroborates these operational bottlenecks, identifying production challenges, marketing constraints, high input prices, fodder access, and production trends as the primary drivers shaping sheep farming in the region.

Based on these findings, concrete interventions are required to enhance farm-level efficiency. Extension services and agricultural advisors should provide targeted training on scientific animal nutrition to prevent overfeeding and reduce unnecessary feed costs. Additionally, farm management strategies should prioritize preventive veterinary healthcare over reactive medicine usage to manage expenditures while maintaining animal welfare. Strengthening producer cooperatives is also essential to mitigate high input prices, improve bargaining power in product marketing, and streamline supply chains in the region.

This research is subject to certain limitations that should be addressed in future studies. The reliance on cross-sectional data from the TRA2 region limits the generalizability of the findings across different agro-ecological zones and time periods. Future research could utilize panel data to analyze efficiency dynamics over time. Furthermore, incorporating environmental efficiency indicators and detailed feed quality parameters into future analytical frameworks would provide a more holistic understanding of sustainable sheep production systems.

## Figures and Tables

**Table 1:** Sample size.

Class Limits (Head)	Stratum	Frequency (Nh)	Standard Deviation (Sh)	Variance (Sh^2^)	Mean (Number of Sheep)	Nh × Sh	Nh × Sh^2^	Sample Size (n)
1–100	I	8763	23.18	537.24	57.23	203,112	4,707,811	22
101–250	II	5606	42.86	1836.60	161.15	240,249	10,296,003	25
251–750	III	3550	125.47	15,743.34	405.35	445,427	55,888,840	47
751+	IV	612	680.75	463,415.02	1179.69	416,617	283,609,995	44
		18,531	262.53	68,922.38	192.43	1,305,404	354,502,649	138

**Table 2:** Distribution of the sample farm size by provinces.

Provinces	Sample Size (n)
Ardahan	4
Kars	20
Ağrı	56
Iğdır	58
TRA2 Region Total	138

**Table 3:** Variables used in the efficiency analyses.

Variables	Minimum	Maximum	Average	Standard Deviation
GPV (USD/LU)	140.20	4236.65	1431.83	643.63
Roughage quantity (kg/LU)	82.12	6694.56	1689.16	1485.06
Concentrated quantity (kg/LU)	1.00	4588.01	574.18	748.07
Family labour (hours/LU)	16.99	1142.86	168.80	197.92
Medication costs (USD/LU)	3.24	169.91	33.10	27.59

**Table 4:** Efficiency results of the enterprises.

Stratum	Efficiency Source			
	Technical Efficiency (SFA)	Technical Efficiency (DEA)	Allocative Efficiency	Economic Efficiency
I	0.598	0.741	0.101	0.072
II	0.569	0.653	0.185	0.124
III	0.542	0.609	0.421	0.272
IV	0.596	0.668	0.497	0.319
Mean	0.573	0.657	0.351	0.228

**Table 5:** Distribution of efficiency scores for enterprises in stratum I.

Number Enterprises	Efficiency Source			
	Technical Efficiency (SFA)	Technical Efficiency (DEA)	Allocative Efficiency	Economic Efficiency
	N			
1.000	0	7	0	0
0.751–0.999	5	5	0	0
0.501–0.750	10	6	0	0
0.251–0.500	6	4	1	0
0.001–0.250	1	0	21	22
Total	22	22	22	22
	**Ratio (%)**			
1.000	0.00	31.82	0.00	0.00
0.751–0.999	22.73	22.73	0.00	0.00
0.501–0.750	45.45	27.27	0.00	0.00
0.251–0.500	27.27	18.18	4.55	0.00
0.001–0.250	4.55	0.00	95.45	100.00
Total	100.00	100.00	100.00	100.00

**Table 6:** Distribution of efficiency scores for enterprises in stratum II.

Number Enterprises	Efficiency Source			
	Technical Efficiency (SFA)	Technical Efficiency (DEA)	Allocative Efficiency	Economic Efficiency
	N			
1.000	0	3	0	0
0.751–0.999	4	5	0	0
0.501–0.750	12	11	1	1
0.251–0.500	8	6	5	3
0.001–0.250	1	0	19	21
Total	25	25	25	25
	**Ratio (%)**			
1.000	0.00	12.00	0.00	0.00
0.751–0.999	16.00	20.00	0.00	0.00
0.501–0.750	48.00	44.00	4.00	4.00
0.251–0.500	32.00	24.00	20.00	12.00
0.001–0.250	4.00	0.00	76.00	84.00
Total	100.00	100.00	100.00	100.00

**Table 7:** Distribution of efficiency scores for enterprises in stratum III.

Number Enterprises	Efficiency Source			
	Technical Efficiency (SFA)	Technical Efficiency (DEA)	Allocative Efficiency	Economic Efficiency
	N			
1.000	0	8	0	0
0.751–0.999	10	6	5	2
0.501–0.750	16	13	14	6
0.251–0.500	18	19	15	10
0.001–0.250	3	1	13	29
Total	47	47	47	47
	**Ratio (%)**			
1.000	0.00	17.02	0.00	0.00
0.751–0.999	21.28	12.77	10.64	4.26
0.501–0.750	34.04	27.66	29.79	12.77
0.251–0.500	38.30	40.43	31.91	21.28
0.001–0.250	6.38	213	27.66	61.70
Total	100.00	100.00	100.00	100.00

**Table 8:** Distribution of efficiency scores for enterprises in stratum IV.

Number Enterprises	Efficiency Source			
	Technical Efficiency (SFA)	Technical Efficiency (DEA)	Allocative Efficiency	Economic Efficiency
	N			
1.000	0	10	0	0
0.751–0.999	18	8	5	0
0.501–0.750	10	11	18	6
0.251–0.500	11	10	15	25
0.001–0.250	5	5	6	13
Total	44	44	44	44
	**Ratio (%)**			
1.000	0.00	22.73	000	0.00
0.751–0.999	40.91	18.18	11,36	0.00
0.501–0.750	22.73	25.00	40.91	13.64
0.251–0.500	25.00	22.73	34.09	56.82
0.001–0.250	11.36	11.36	13.64	29.55
Total	100.00	100.00	100.00	100.00

**Table 9:** Variables used in the Tobit and inefficiency analyses.

Variables	Minimum	Maximum	Average	Standard Deviation
Producer age (years)	27.00	77.00	51.20	11.34
Sheep farming experience (years)	1.00	50.00	17.44	8.40
Producer education (years)	1.00	11.00	5.04	1.96
Household size (persons)	2.00	9.00	6.67	1.85
Number of feedings	2.00	5.00	2.62	0.53

**Table 10:** Tobit regression analysis results.

Variables	Coefficient	Standard Error	*p* Value
**Tobit Analysis Results**			
Constant	1.132 ***	0.172	0.000
Sheep farming experience	−0.004	0.003	0.216
Household size	−0.016	0.012	0.171
Farmer age	0.001	0.002	0.769
Farmer education duration	−0.013	0.011	0.233
Number of feedings in the enterprise	−0.106 ***	0.041	0.009
**Statistical Results of the Model**			
Scale: c (7)	0.247	0.015	0.000
R^2^	0.092		
Adjusted R^2^	0.050		

*** Denotes statistical significance at the 1% level.

**Table 11:** Parameter estimations of the stochastic frontier analysis model.

Variables	Coefficient	Standard Error	t Value
**Stochastic Frontier Results**			
Constant	6.731 ***	2.072	3.248
Ln (Roughage)	0.047	0.134	0.353
Ln (Concentrate feed)	0.025	0.022	1.171
Ln (Family labour)	0.195	0.172	1.133
Ln (Veterinary medicine)	0.136 *	0.099	1.377
Ln (Number of sheep)	0.280 *	0.217	1.291
**Inefficiency Effects Model**			
Constant	−0.539	2.109	−0.255
Sheep farming experience	−0.143	0.236	−0.605
Household size	0.426	0.455	0.935
Farmer age	−0.086	0.496	−0.172
Farmer education duration	0.002	0.106	0.017
Number of feedings in the enterprise	0.740 *	0.541	1.368
**Statistical Results of the Model**			
Returns to scale	0.644 (IRS)		
Sigma squared	0.511 *	0.388	1.318
Gamma	0.949 ***	0.052	18.225
Log likelihood (H_0_)	−99.224		
Log likelihood function (H_1_)	−88.894		
Likelihood ratio (LR) test	20.659		
Mean technical efficiency	0.573		

***,* Denote statistical significance at the 10% and 1% levels, respectively. IRS: Increasing returns to scale.

**Table 12:** Functional analysis of sheep farming.

Variables	Elasticities	Standard Error	t Value
Constant	6.731 ***	2.072	3.248
X_1_ (Roughage)	0.047	0.134	0.353
X_2_ (Concentrate feed)	0.025	0.022	1.171
X_3_ (Family labour)	0.195	0.172	1.133
X_4_ (Veterinary medicine cost)	0.136 *	0.099	1.377
X_5_ (Number of sheep)	0.280 *	0.217	1.291

***,* Denote statistical significance at the 10% and 1% levels, respectively.

**Table 13:** Factor analysis of sheep farming.

Data Code.	Variables	Explanation
B_1_	Frequency of internet usage	1 = Every day 2 = 1–2 times a week 3 = More than 3 times a week 4 = Once a month 5 = Once a year
B_2_	Importance of written information sources in sheep farming	1 = Not important at all 2 = Slightly important 3 = Undecided 4 = Important 5 = Very important
B_3_	Problems with disease management in sheep farming	
B_4_	Problems with securing shepherds in sheep farming	
B_5_	Problems with livestock and product transportation in sheep farming	
B_6_	Problems with obtaining suitable credit/loans in sheep farming	
B_7_	High input prices in sheep farming	
B_8_	Low product prices in sheep farming	
B_9_	Experiencing difficulties in procuring high-quality fodder in sheep farming	1 = Definitely yes 2 = To a great extent 3 = Undecided 4 = Slightly 5 = Definitely no
B_10_	High input prices affecting production decisions in sheep farming	
B_11_	Experiencing marketing problems in sheep farming	
B_12_	Experiencing problems due to insufficient infrastructure in sheep farming	
B_13_	Knowledge level regarding the marketing of sheep products	1 = Very low 2 = Low 3 = Moderate 4 = High 5 = Very high
B_14_	Intention to continue sheep farming activities	

**Table 14:** KMO ve Bartlett’s tests.

KMO measure of sampling adequacy		0.759
Bartlett’s tests	Approx. Chi-square	852.872
	df	91
	Sig.	0.000

**Table 15:** Number of factors based on eigenvalue statistics and total variance explained.

Factor	Initial Eigenvalues			Rotation Sums of Squared Loadings		
	Total	Variance (%)	Cumulative (%)	Total	Variance (%)	Cumulative (%)
1	4.44	31.72	31.72	4.42	31.55	31.55
2	1.91	13.67	45.38	1.91	13.67	45.22
3	1.85	13.19	58.57	1.60	11.39	56.61
4	1.17	8.35	66.92	1.44	10.31	66.92
…	…	…	…			
…	…	…	…			
14	0.11	0.78	100.00			

**Table 16:** Common variance.

Variables	Initial	Extraction
B_1_	1.000	0.651
B_2_	1.000	0.518
B_3_	1.000	0.679
B_4_	1.000	0.653
B_5_	1.000	0.800
B_6_	1.000	0.801
B_7_	1.000	0.643
B_8_	1.000	0.623
B_9_	1.000	0.665
B_10_	1.000	0.639
B_11_	1.000	0.711
B_12_	1.000	0.591
B_13_	1.000	0.706

**Table 17:** Rotated factor matrix.

Variables	Factor			
	1	2	3	4
B_6_	0.886			
B_5_	0.873			
B_7_	0.794			
B_4_	0.787			
B_3_	0.784			
B_8_	0.749			
B_2_	0.630			
B_11_		0.833		
B_10_		0.776		
B_12_		0.672	0.352	
B_1_			0.805	
B_9_			0.780	
B_13_				0.829
B_14_				0.795

Extraction Method: PCA; Rotation Method: Varimax with Kaiser Normalization; Rotation converged in 5 iterations.

## Data Availability

The data analyzed in this study are not publicly available due to participant privacy and ethical restrictions. However, they may be made available by the corresponding author upon reasonable request and in accordance with relevant ethical guidelines.

## Abbreviations

The following abbreviations are used in this manuscript:

AE	Allocative Efficiency
BBHB	Livestock Unit
da	Decare
DEA	Data envelopment analysis
EE	Economic Efficiency
FAO	Food and Agriculture Organization
GPV	Gross Production Value
IRS	Increasing returns to scale
KMO	Kaiser-Meyer-Olkin
LR	Likelihood Ratio
LU	Livestock Units
PCA	Principal component analysis
SD	Standard deviation
SFA	Stochastic frontier analysis
TE	Technical efficiency
TRY	Turkish lira
TURKSTAT	Turkish Statistical Institute

## References

[1] Acharya R.M., Timon V.M., Hanrahan J.P. (1985). Small ruminant production in Arid and Semi-Arid Asia. Small Ruminant Production in the Developing Countries.

[2] Joy A., Dunshea F.R., Leury B.J., Clarke I.J., DiGiacomo K., Chauhan S.S. (2020). Resilience of small ruminants to climate change and increased environmental temperature: A review. Animals.

[3] Recha J., Demissie T., Haile A., Mengistu T.G., Makonnen B.T., Ambaw G., Barungi J., Magagula F., Taapopi M., Gitau A. (2025). Catalysing the Scale-Up of Integrated Small Ruminant Value Chains in Eastern and Southern Africa.

[4] Udo H.M.J., Aklilu H.A., Phong L.T., Bosma R.H., Budisatria I.G.S., Patil B.R., Samdup T., Bebe B.O. (2011). Impact of intensification of different types of livestock production in smallholder crop-livestock systems. Livest. Sci..

[5] Günlü A., Mat B., Erdem H., Çiftçi E., Işık M.K., Yorgancılar M.Ü. (2021). The place and importance of sheep and goat breeding in the economy of Türkiye. Sheep and Goat Health and Breeding in the Prevention of Lamb and Kid Losses.

[6] TİGEM (General Directorate of Agricultural Enterprises) (2022). Livestock Sector Report.

[7] Patil V.M. (2008). Sheep and Goat Production Management.

[8] Arslan M., Kurğa C., Borazan A. (2017). Current Status and Strategic Action Plan for Small Ruminant Livestock in the TRA2 Region.

[9] FAOSTAT, Food and Agriculture Organization of the United Nations (2025). https://www.fao.org/faostat/en/#data/QCL.

[10] Ministry of Agriculture and Forestry (HAYGEM) (2021). Sheep Breeding.

[11] Çiçek A., Ayyıldız M., Erdal G., Erdal H. (2022). The importance and economic analysis of sheep breeding in Türkiye. MAS J. Appl. Sci..

[12] Alkan M. (2010). The bitter end of small ruminant livestock. Çiftlik Dergisi.

[13] Eastern Anatolia Development Agency (DAKA) (2012). Small Ruminant Livestock Workshop Report.

[14] Nix J. (1988). The economics of sheep production. Br. Vet. J..

[15] Şirikçi B.S. (2026). Beyond technical efficiency: Structural disconnect between managerial resource use and sustainability in water buffalo farming in Türkiye. Animals.

[16] Yılmaz Ş.G. (2019). Socio-Economic Analysis and Efficiency of Small Ruminant Farms: The Case of Western Mediterranean Region. Ph.D. Dissertation.

[17] Çiçek A., Erkan O. (1996). Research and Sampling Methods in Agricultural Economics.

[18] Yolalan R. (1993). Measurement of Relative Efficiency Between Businesses.

[19] Savickienė R., Namiotko V. (2026). Intensification and technical efficiency in dairy farming: Evidence from the Baltic States and Poland. Sustainability.

[20] Farrell M.J. (1957). The measurement of productive efficiency. J. R. Stat. Soc. Ser. A Gen..

[21] Bailey D., Hubert T. (1990). Productivity Measurement: An International Review of Concepts, Techniques, Programmes, and Currents Issues.

[22] Berk A., Güney O.İ., Sangün L. (2022). Measurement of resource use efficiency in corn production: A two-stage data envelopment analysis approach in Turkey. Cienc. Rural..

[23] Sickles R.C., Zelenyuk V. (2019). Measurement of Productivity and Efficiency: Theory and Practice.

[24] Sultana S., Hossain M.M., Haque M.N. (2023). Estimating the potato farming efficiency: A comparative study between stochastic frontier analysis and data envelopment analysis. PLoS ONE.

[25] Kumbhakar S.C., Lovell K.C.A. (2000). Stochastic Frontier Analysis.

[26] Munde P.V., Shendage P.N., Rahane J.J., Hile R.B., Dhok A.A. (2025). Efficiency Analysis of Member and Non-Member Farmers of FPOs Using Data Envelopment Analysis. J. Exp. Agric. Int..

[27] Asmara R., Fahriyah F., Sulistiowati S.E. (2025). Soybean Farmer Performance (*Glycine max* L.) From The Perspective of Technical Efficiency Through Comparison of Parametric (SFA) And Non-Parametric (DEA) Approaches. Proceedings of the 2024 Brawijaya International Conference (BIC 2024).

[28] Kadakoğlu B., Karlı B. (2022). Technical efficiency of potato production in Turkey by Stochastic Frontier Analysis. Cust. Agronegocio.

[29] Charnes A., Cooper W.W., Rhodes E. (1978). Measuring the efficiency of decision-making units. Eur. J. Oper. Res..

[30] Banker R.D., Thrall R.M. (1992). Estimation of returns to scale using data envelopment analysis. Eur. J. Oper. Res..

[31] Novaković T., Novaković D., Milić D., Tomaš Simin M., Radišić M., Radišić M., Nikolić S., Mihajlović M. (2025). Assessment of technical and eco-efficiency of dairy farms in the republic of Serbia: Towards the implementation of a circular economy. Agriculture.

[32] Talluri S. (2000). Data envelopment analysis: Models and extensions. Decis. Line.

[33] Kocakoç İ.D. (2003). Use of analytic hierarchy process in determining weight restrictions in Data Envelopment Analysis. Dokuz Eylül Univ. J. Fac. Econ. Adm. Sci..

[34] Cappella M.T., Caracciolo F., Blasi E. (2026). A stochastic frontier approach to nitrogen use and efficiency in soft wheat cultivation. Precis. Agric..

[35] Aigner D., Lovell C.K., Schmidt P. (1977). Formulation and estimation of stochastic frontier production function models. J. Econom..

[36] Battese G.E., Corra G.S. (1977). Estimation of a production frontier model: With application to the pastoral zone of Eastern Australia. Aust. J. Agric. Econ..

[37] Meeusen W., Van Den Broeck J. (1977). Efficiency estimation from Cobb-Douglas production functions with composed error. Int. Econ. Rev..

[38] Berger A.N., Humphrey D.B. (1997). Efficiency of financial institutions: International survey and directions for future research. Eur. J. Oper. Res..

[39] Kutlar A. (2017). Applications of Logistic Regression (Logit), Probit and Tobit Models with EViews and SPSS.

[40] Emir B., Çolak E., Öner K.S. (2018). On the performance of estimators in standard tobit model under different optimization techniques and sample sizes: A simulation study. Osman. J. Med..

[41] Greene W.H. (2003). Econometric Analysis.

[42] Burbano Pulles M.R., Cuadrado Merlo J.B. (2026). A Data-driven framework for assessing sustainability-oriented research models in higher education institutions. Sustainability.

[43] Dağıstan E., Koç B., Gül A., Gül M. (2008). Factor analysis of sheep production activity: The case of Central-South Anatolia. Tarım Bilim. Derg..

[44] Graffelman J., Balakrishnan N., Colton T., Everitt B., Piegorsch B., Ruggeri F., Teugels J.L. (2014). Factor Analysis. Wiley StatsRef: Statistics Reference Online.

[45] Joseph F., Hair J.R., Rolph E.A., Ronald L.T., William C.B. (1992). Multivariate Data Analysis.

[46] Sharma P., Singh A.K., Leiva V., Martin-Barreiro C., Cabezas X. (2022). Modern multivariate statistical methods for evaluating the impact of whatsApp on academic performance: Methodology and case study in India. Appl. Sci..

[47] Fernández Álvarez J., León Jurado J.M., Navas González F.J., Iglesias Pastrana C., Delgado Bermejo J.V. (2020). Optimization and validation of a linear appraisal scoring system for milk production-linked zoometric traits in Murciano-Granadina dairy goats and bucks. Appl. Sci..

[48] Ye N., Xu Y., Zhang R. (2025). Farmers’ farmland quality protection behavior: Influencing factors and policy implications in eastern coastal China. Front. Sustain. Resour. Manag..

[49] (2026). Merkez Bankası The Central Bank of the Republic of Türkiye Electronic Data Delivery System. https://evds3.tcmb.gov.tr/index.php?/evds/serieMarket/#collapse_2.

[50] Al-Tamimi A.A.A., Abdullah D.F. (2025). Economic analysis for achieving allocative-technical efficiency in sustainable agricultural projects (calf fattening farm as a model). AIJANS.

[51] Coelli T.J., Rao D.S.P., O’Donnell C.J., Battese G.E. (2005). An Introduction to Efficiency and Productivity Analysis.

[52] Parlakay O., Alemdar T. (2011). Technical and economic efficiency of peanut production in Turkey. TJAE.

[53] Hair J.F., Anderson R.E., Tatham R.L., Black W.C. (1998). Multivariate Data Analysis.

[54] Flanagan-Bórquez A., Soriano-Soriano G., Jiménez-Quinteros P., Carvajal-Araneda K., Escalante-Barrios E. (2026). Development and validation of subscales for assessing first-generation university students’ experiences. J. New Approaches Educ. Res..

[55] Sokratous S., Ragkos A., Arsenos G., Theodoridis A. (2025). Efficiency analysis of sheep farms in Cyprus. Animals.

[56] Theodoridis A., Ragkos A., Roustemis D., Galanopoulos K., Abas Z., Sinapis E. (2012). Assessing technical efficiency of chios sheep farms with data envelopment analysis. Small Rumin. Res..

[57] Toro-Mujica P., Garcia A., Gomez-Castro A.G., Acero R., Perea J., Rodriguez-Estevez V., Aguilar C., Vera R. (2011). Technical efficiency and viability of organic dairy sheep farming systems in a traditional area for sheep production in Spain. Small Rumin. Res..

[58] Şirikçi B.S., Gül M. (2026). Cost and Profitability of Raising Domestic Cattle in Türkiye. Sci. Pap. Ser. Manag. Econ. Eng. Agric. Rural Dev..

[59] Gül M., Demircan V., Yılmaz H., Yılmaz H. (2016). Technical efficiency of goat farming in Turkey: A case study of Isparta province. Rev. Bras. Zootec..

[60] Dalgıç A. (2018). Koyunculuk Üretim Faaliyetinin Ekonomik Analizi: Isparta İli Örneği. Master’s Thesis.

[61] Kaliba A.R., Engle C.R. Cost efficiency of catfish farms in Chicot County, Arkansas: The impact of extension purposes. Proceedings of the Southern Agricultural Economics Association Annual Meeting.

[62] Battese G.E., Coelli T.J. (1995). A model for technical inefficiency effects in a stochastic frontier production function for panel data. Empir. Econ..

[63] Külekçi M., Bayram B. (2012). Buzağıların beslenmelerinde etkinliğin belirlenmesi stokastik sınır analizi uygulaması. Harran Tarım Gıda Bilim. Derg..

[64] Fathelrahman E., Sherif S., Hoag D.L.K. (2014). Small ruminant production system efficiency under Abu-Dhabi, United Arab Emirates arid land conditions. Agriculture.

